# Supplementary material for: Rank‐based Bayesian variable selection for genome‐wide transcriptomic analyses
Source: Stat Med. 2022 Jul 18;41(23):4532–53. doi: 10.1002/sim.9524 (PMC9796757; doi:10.1002/sim.9524)
Supplement: Supplementary file 1 — Figure S1 Top‐rank data generating process with ntrue∗=nguess∗=8. Each panel displays the probability of items being ranked 1,…,n∗. From left to right, l=1,2,3, and from top to bottom L=1,2,3; α=10, the true rank is indicated as a rainbow grid, and the bar plot indicates the number of iterations the item was given a rank in the MCMC Figure S2 Top‐rank data generating process with ntrue∗=8, nguess∗=4. Each panel displays the probability of items being ranked 1,…,n∗−4. From left to right, l=1,2,3, and from top to bottom L=1,2,3; α=3, the true rank is indicated as a rainbow grid, and the bar plot indicates the number of iterations the item was given a rank Figure S3 Top‐rank data generating process with ntrue∗=8, nguess∗=4. Each panel displays the probability of items being ranked 1,…,n∗−4. From left to right, l=1,2,3, and from top to bottom L=1,2,3; α=10, the true rank is indicated as a rainbow grid, and the bar plot indicates the number of iterations the item was given a rank Figure S4 Top‐rank data generating process with ntrue∗=8, nguess∗=12. Each panel displays the probability of items being ranked 1,…,n∗+4. From left to right, l=1,2,3, and from top to bottom L=1,2,3; α=3, the true rank is indicated as a rainbow grid, and the bar plot indicates the number of iterations the item was given a rank in the MCMC Figure S5 Top‐rank data generating process with ntrue∗=8, nguess∗=12. Each panel displays the probability of items being ranked 1,…,n∗+4. From left to right, l=1,2,3, and from top to bottom L=1,2,3; α=10, the true rank is indicated as a rainbow grid, and the bar plot indicates the number of iterations the item was given a rank in the MCMC Figure S6 Top‐rank data generating process with n∗=50. Each panel displays the trace plot of the top 15 items being ranked 1,…,n∗ from left to right: L=1 and L=2 with α=10 and l=round(n∗/5) Figure S7 Top‐rank data generating process with n∗=50. Proportion of correct items in the posterior distribution of 𝒜∗ by varying L. α=10 a [file SIM-41-4532-s001.pdf]

Supplementary material to the paper: Rank-based Bayesian variable selection for genome-wide transcriptomic analyses

Emilie Eliseussen, Thomas Fleischer and Valeria Vitelli

S1.

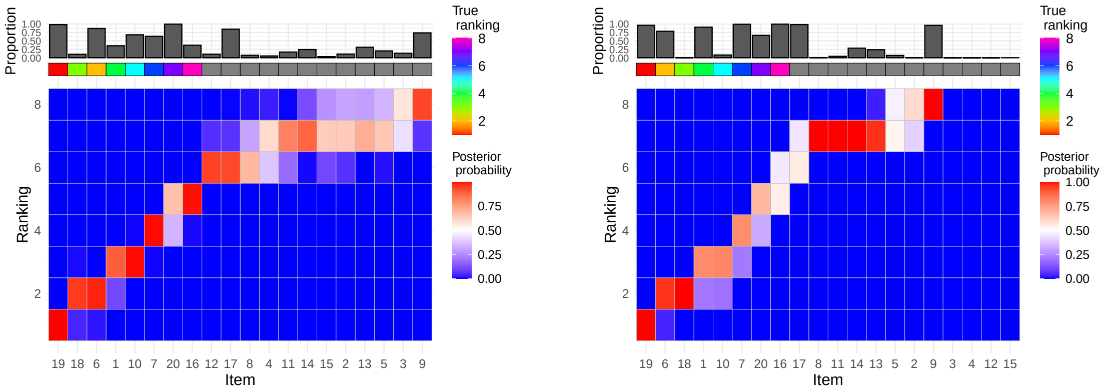

Figure 1: Results from the top-rank simulation experiments described in Section 3.1: heatplots of the marginal posterior distribution of $\boldsymbol{\rho}$, where the items have been ordered according to $\hat{\boldsymbol{\rho}}_{\mathcal{A}*}$ on the x-axis. Left $\alpha = 3$, right $\alpha = 10$. The rainbow grid indicates the true $\boldsymbol{\rho}_{\mathcal{A}*}$, and the bar plot indicates the proportion of times the items were selected in $\mathcal{A}^*$ over all MCMC iterations. $n = 20$, $N = 50$, $n^* = 8$, $L = 1$ and $l = 2$.

S2.

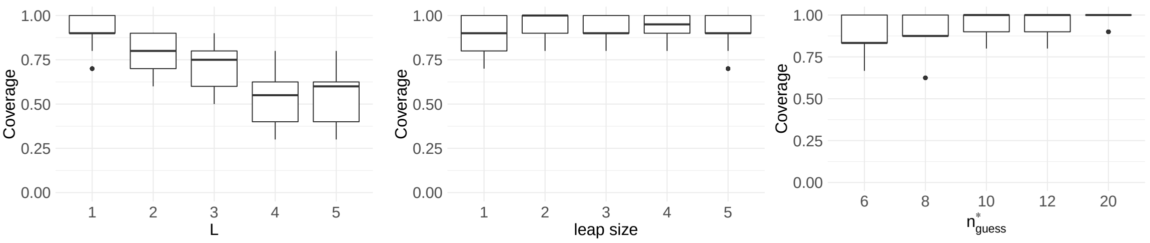

Figure 2: Results from the sensitivity study described in Section 3.2: each panel displays boxplots of the proportion of correct items selected, $\hat{p}$, over 20 runs on different datasets, for varying values of the tuning parameters on the x-axis. From left to right: varying L, l and n^*_{guess}, respectively. $n = 100$, $N = 10$, $n^* = 10$, $\alpha = 5$, $M = 5000$.

S3.

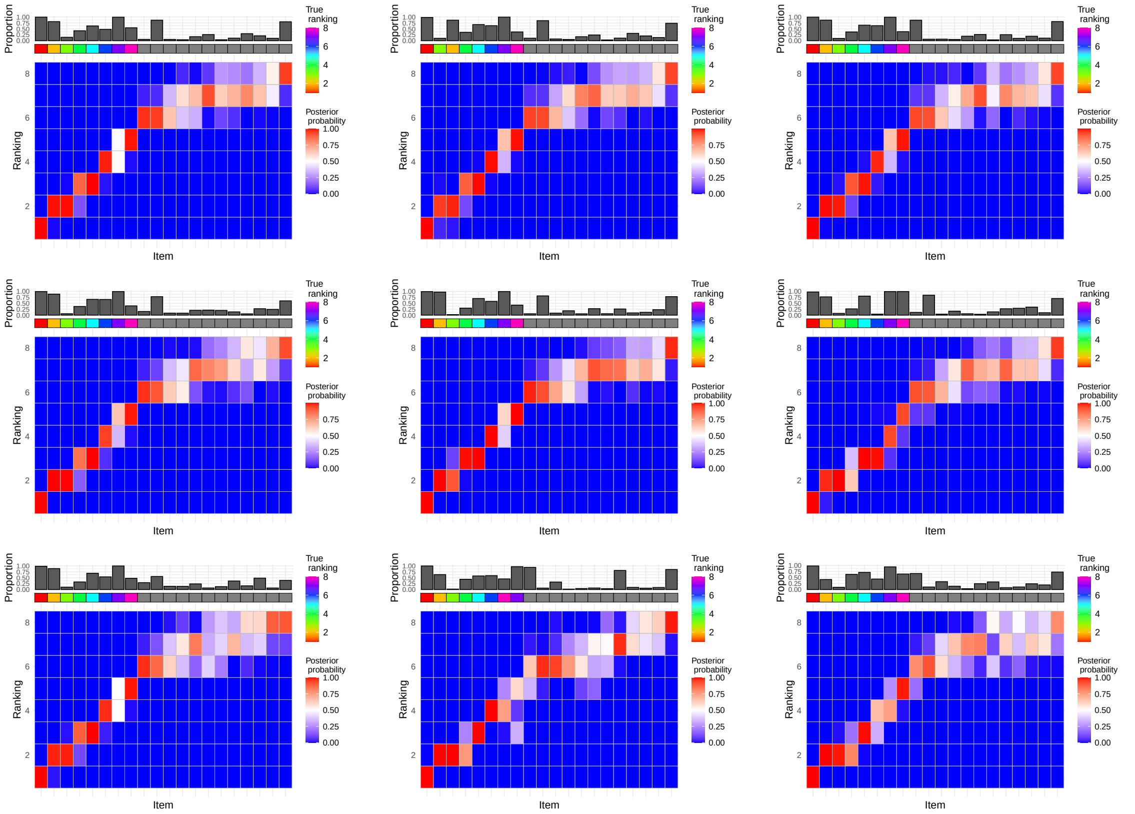

Figure 3: Results from the top-rank simulation experiments described in Section 3.2: each panel displays the marginal posterior distribution of $\boldsymbol{\rho}$, where the items have been ordered according to $\hat{\boldsymbol{\rho}}_{\mathcal{A}*}$ on the x-axis. From left to right $l = 1, 2, 3$, and from top to bottom $L = 1, 2, 3$. The rainbow grid indicates the true $\boldsymbol{\rho}_{\mathcal{A}*}$, and the bar plot indicates the proportion of times the items were selected in $\mathcal{A}^*$ over all MCMC iterations. $n^* = 8$, $n = 20$, $N = 50$, $\alpha = 3$.

S4.

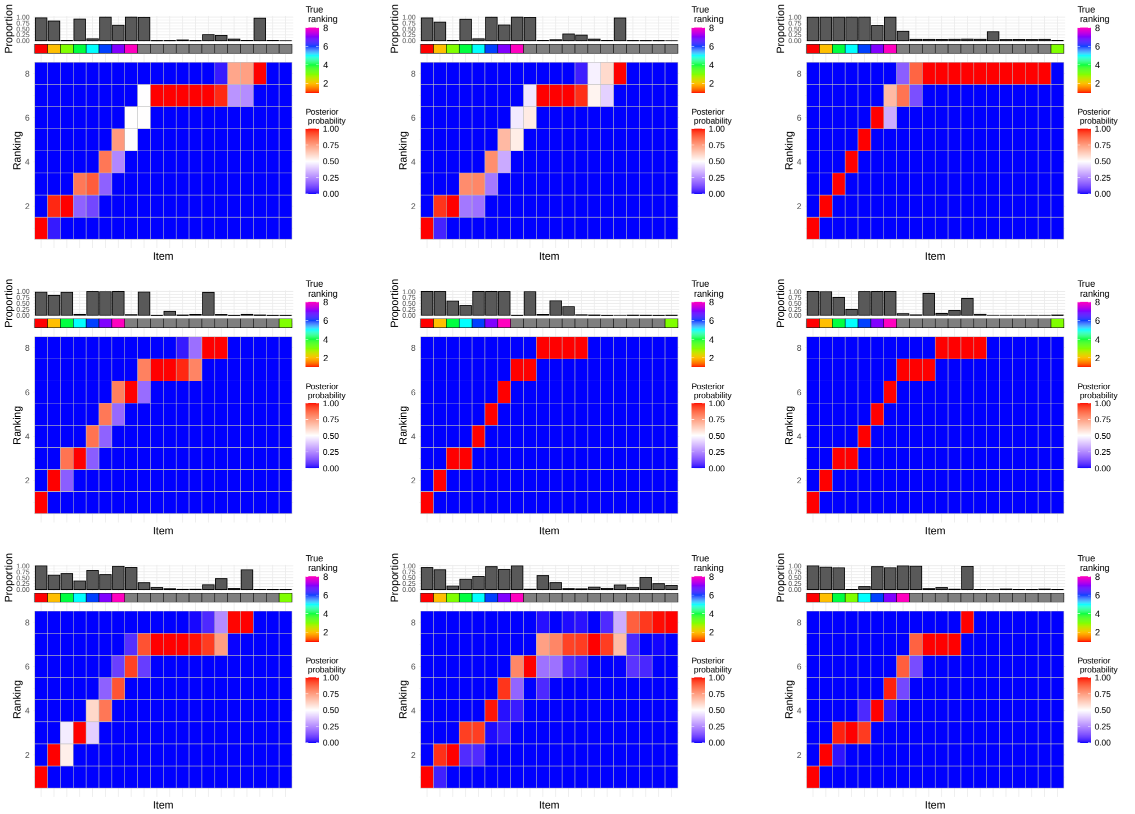

Figure 4: Results from the top-rank simulation experiments described in Section 3.2: each panel displays the marginal posterior distribution of $\boldsymbol{\rho}$, where the items have been ordered according to $\hat{\boldsymbol{\rho}}_{\mathcal{A}*}$ on the x-axis. From left to right $l = 1, 2, 3$, and from top to bottom $L = 1, 2, 3$. The rainbow grid indicates the true $\boldsymbol{\rho}_{\mathcal{A}*}$, and the bar plot indicates the proportion of times the items were selected in $\mathcal{A}^*$ over all MCMC iterations. $n^* = 8$, $n = 20$, $N = 50$, $\alpha = 10$.

S5.

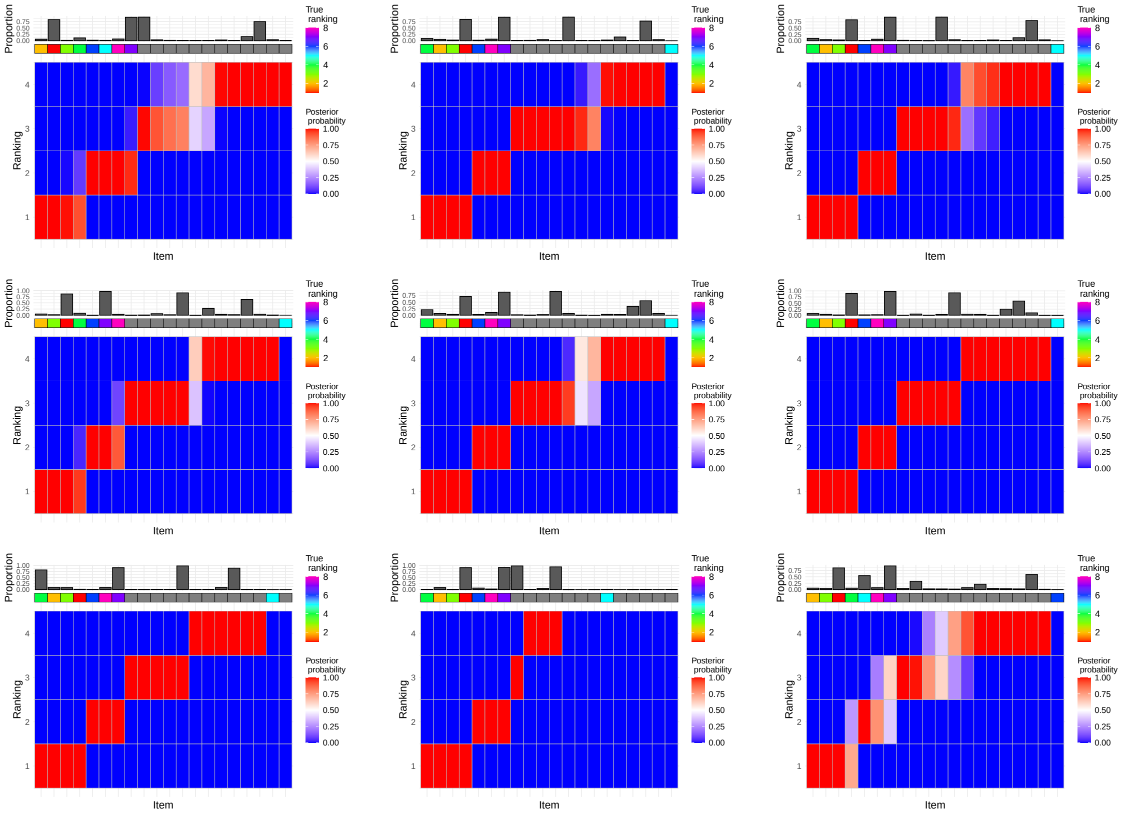

Figure 5: Results from the top-rank simulation experiments described in Section 3.2 with $n^*_{\text{true}} = 8$, $n^*_{\text{guess}} = 4$: each panel displays the marginal posterior distribution of $\boldsymbol{\rho}$, where the items have been ordered according to $\hat{\boldsymbol{\rho}}_{\mathcal{A}*}$ on the x-axis. From left to right $l = 1, 2, 3$, and from top to bottom $L = 1, 2, 3$. The rainbow grid indicates the true $\boldsymbol{\rho}_{\mathcal{A}*}$, and the bar plot indicates the proportion of times the items were selected in $\mathcal{A}^*$ over all MCMC iterations. $n = 20$, $N = 50$, $\alpha = 3$.

S6.

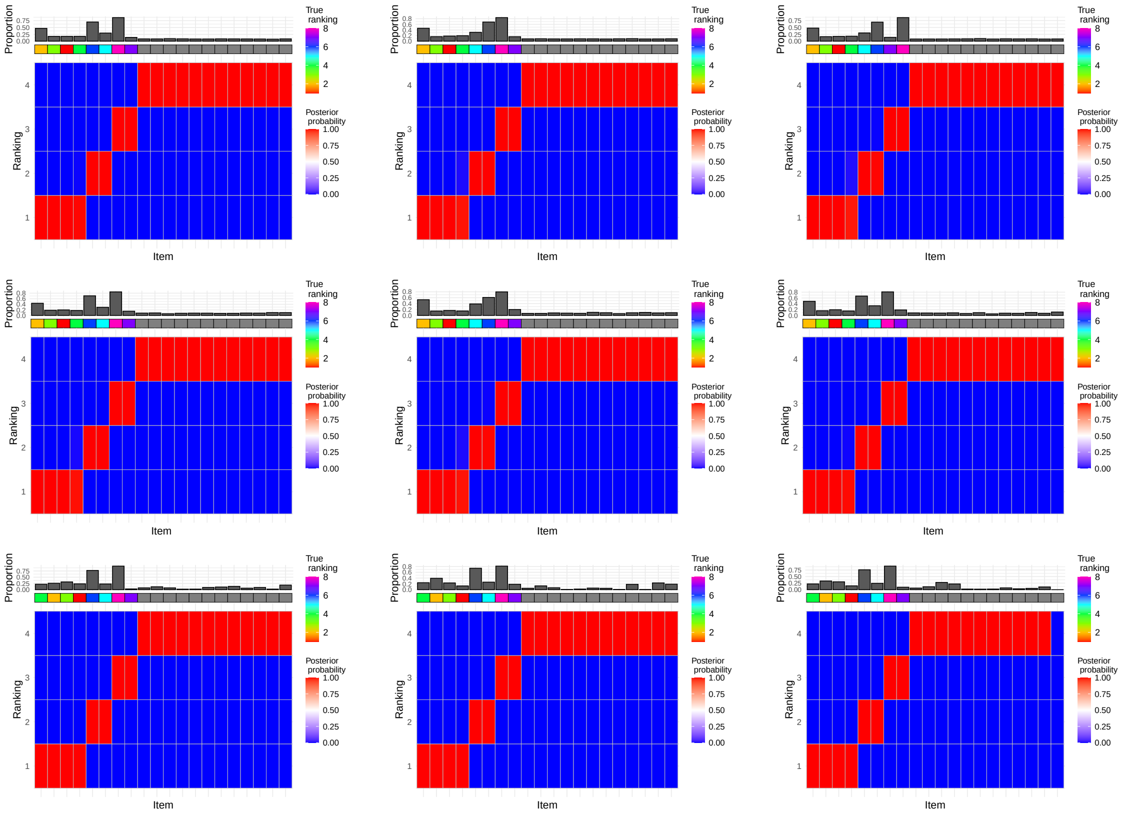

Figure 6: Results from the top-rank simulation experiments described in Section 3.2 with $n^*_{\text{true}} = 8$, $n^*_{\text{guess}} = 4$: each panel displays the marginal posterior distribution of $\boldsymbol{\rho}$, where the items have been ordered according to $\hat{\boldsymbol{\rho}}_{\mathcal{A}*}$ on the x-axis. From left to right $l = 1, 2, 3$, and from top to bottom $L = 1, 2, 3$. The rainbow grid indicates the true $\boldsymbol{\rho}_{\mathcal{A}*}$, and the bar plot indicates the proportion of times the items were selected in $\mathcal{A}^*$ over all MCMC iterations. $n = 20$, $N = 50$, $\alpha = 10$.

S7.

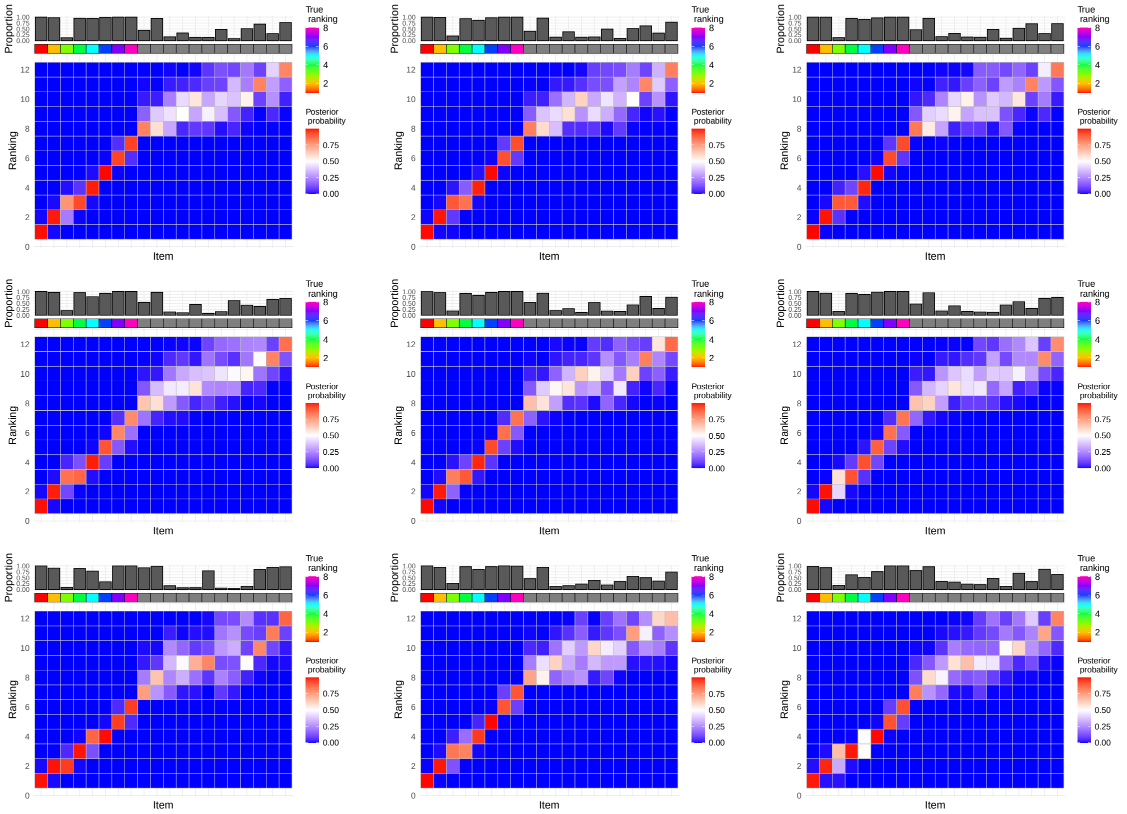

Figure 7: Results from the top-rank simulation experiments described in Section 3.2 with $n^*_{\text{true}} = 8$, $n^*_{\text{guess}} = 12$: each panel displays the marginal posterior distribution of $\boldsymbol{\rho}$, where the items have been ordered according to $\hat{\boldsymbol{\rho}}_{\mathcal{A}*}$ on the x-axis. From left to right $l = 1, 2, 3$, and from top to bottom $L = 1, 2, 3$. The rainbow grid indicates the true $\boldsymbol{\rho}_{\mathcal{A}*}$, and the bar plot indicates the proportion of times the items were selected in $\mathcal{A}^*$ over all MCMC iterations. $n = 20$, $N = 50$, $\alpha = 3$.

S8.

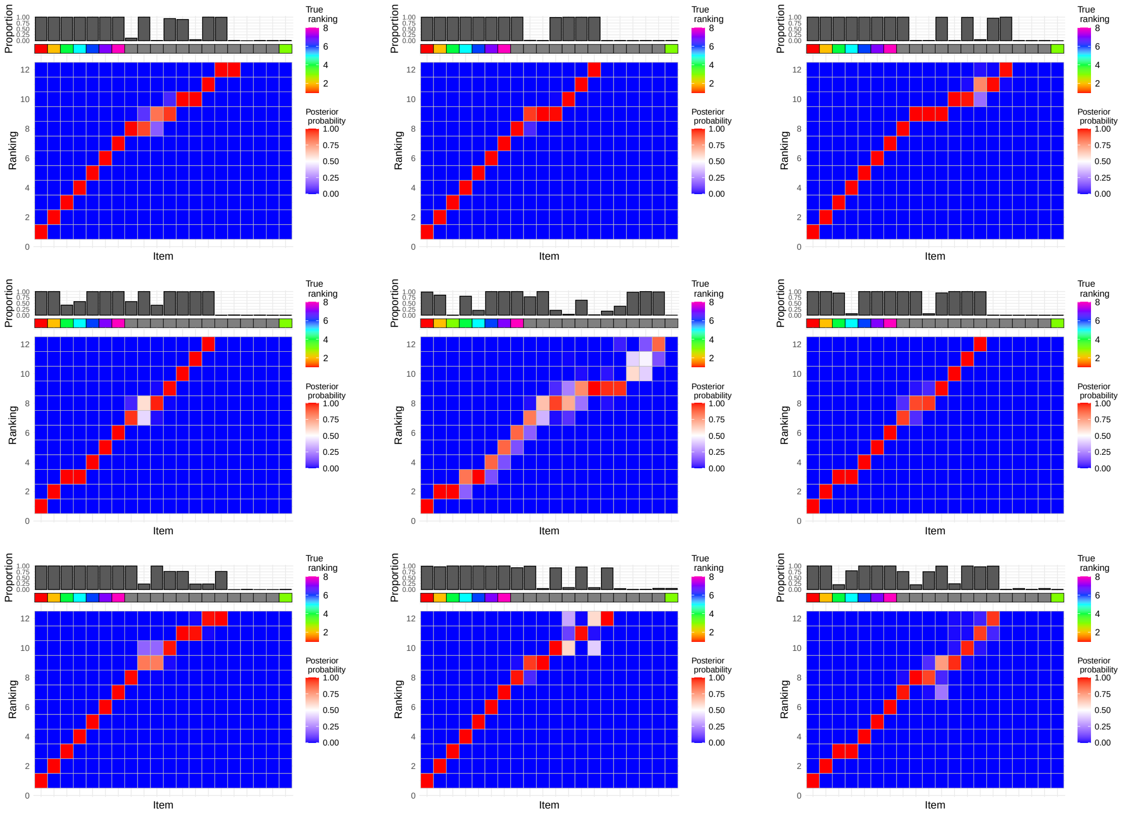

Figure 8: Results from the top-rank simulation experiments described in Section 3.2 with $n^*_{\text{true}} = 8$, $n^*_{\text{guess}} = 12$: each panel displays heatplots of the marginal posterior distribution of $\boldsymbol{\rho}$, where the items have been ordered according to $\hat{\boldsymbol{\rho}}_{\mathcal{A}*}$ on the x-axis. From left to right $l = 1, 2, 3$, and from top to bottom $L = 1, 2, 3$. The rainbow grid indicates the true $\boldsymbol{\rho}_{\mathcal{A}*}$, and the bar plot indicates the proportion of times the items were selected in $\mathcal{A}^*$ over all MCMC iterations. $n = 20$, $N = 50$, $\alpha = 10$.

S9.

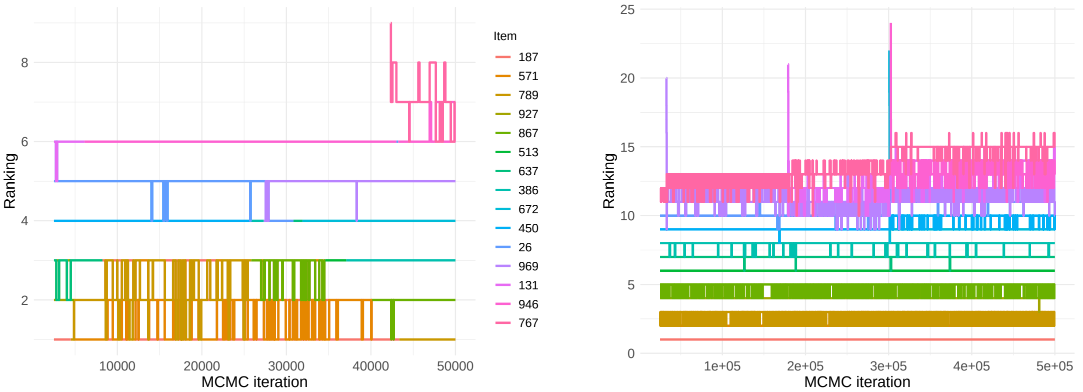

Figure 9: Results from the top-rank simulation experiments described in Section 3.1 with $n^* = 50$. Each panel displays the trace plot of the top-15 items in $\boldsymbol{\rho}_{\mathcal{A}^*}$. From left to right: $L = 1$ and $L = 5$. $n = 1000$, $N = 50$, $\alpha = 10$ and $l = \text{round}(n^*/5)$.

S10.

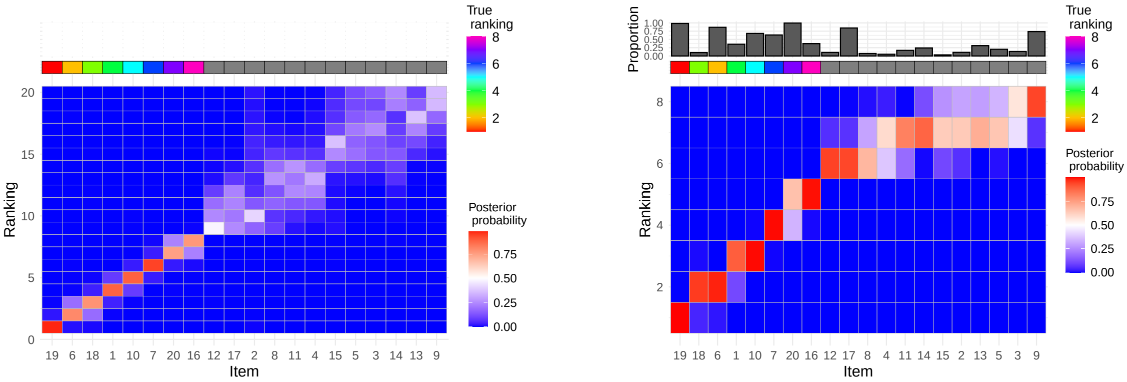

Figure 10: Results from the simulation experiments described in Section 3.4: comparison of the `BayesMallows` model on the complete set of items to lowBMM with $n^* = 8$, $n = 20$, $N = 50$. Heatplots of the marginal posterior distribution of $\boldsymbol{\rho}$, where the items have been ordered according to $\hat{\boldsymbol{\rho}}_{\mathcal{A}^*}$ on the x-axis, for BMM on the left and lowBMM on the right. The rainbow grid indicates the true $\boldsymbol{\rho}_{\mathcal{A}^*}$, and the bar plot indicates the proportion of times the items were selected in $\mathcal{A}^*$ over all MCMC iterations. Parameters for lowBMM: $\alpha = 3$, $L = 1$, $l = 2$.
